# Application of Data Particle Geometrical Divide Algorithms in the Process of Radar Signal Recognition

**DOI:** 10.3390/s23198183

**Published:** 2023-09-30

**Authors:** Janusz Dudczyk, Łukasz Rybak

**Affiliations:** Institute of Telecommunications Systems, Faculty of Electronics, Military University of Technology, 00-908 Warsaw, Poland; lukasz.rybak@wat.edu.pl

**Keywords:** Specific Emitter Identification, radar recognition, radar identification, geometrical divide, data particle, imbalanced data sets, occupancy detection, ELINT

## Abstract

The process of recognising and classifying radar signals and their radiation sources is currently a key element of operational activities in the electromagnetic environment. Systems of this type, called ELINT class systems, are passive solutions that detect, process, and analyse radio-electronic signals, providing distinctive information on the identified emission source in the final stage of data processing. The data processing in the mentioned types of systems is a very sophisticated issue and is based on advanced machine learning algorithms, artificial neural networks, fractal analysis, intra-pulse analysis, unintentional out-of-band emission analysis, and hybrids of these methods. Currently, there is no optimal method that would allow for the unambiguous identification of particular copies of the same type of radar emission source. This article constitutes an attempt to analyse radar signals generated by six radars of the same type under comparable measurement conditions for all six cases. The concept of the SEI module for the ELINT system was proposed in this paper. The main aim was to perform an advanced analysis, the purpose of which was to identify particular copies of those radars. Pioneering in this research is the application of the author’s algorithm for the data particle geometrical divide, which at the moment has no reference in international publication reports. The research revealed that applying the data particle geometrical divide algorithms to the SEI process concerning six copies of the same radar type allows for almost three times better accuracy than a random labelling strategy within approximately one second.

## 1. Introduction

The process of identifying electromagnetic radiation with the use of various electronic sensors, which include radar signals, in North Atlantic Treaty Organization (NATO) countries is described by the acronym ELINT (electronic intelligence) [1]. The idea behind the ELINT class systems is the acquisition, processing, and recognition of radar signals. These are electronic signals that are not used in communication between people; therefore, they do not carry voice messages or text messages [2,3], which are subjects of interest in COMINT (communications intelligence) systems. Thus, it can be concluded that the type of signals that are in the area of interest to the systems of particular classes is a distinctive feature among the set of SIGINT (signal intelligence) solutions.

Existing ELINT class systems are mast-mounted solutions, mounted on land or sea platforms. These solutions significantly limit the space for the acquisition of radio-electronic signals [4]. In terms of the depth of tactical reconnaissance, the distances are from 11 to 19 kilometres. This is mainly conditioned by the technical parameters of the emission source itself (e.g., power of the transmitted signal, characteristics of device operation). An inherent feature of the radio-electronic signal acquisition process is the occurrence of various propagation phenomena during its duration, such as multipath propagation, diffraction, or electromagnetic wave interference [5,6,7]. These phenomena significantly affect the efficiency of the described process. Therefore, it becomes reasonable to adapt ELINT-class systems to unmanned aerial vehicles (UAVs). Solutions of this type are not trivial because, in addition to the radio–electronic signal acquisition process, they must effectively (quasi-online) process data and extract features. As a result, an EDW vector (emitter description word) is created, which describes an emitter. The purpose of the described procedure is to enable the transmission of recorded data, which refers to an emission source, to the UAV command and control station without degrading the capacity of the radio channel used to control the unmanned aerial platform. The described approach enables electromagnetic infiltration, including the acquisition of a radio–electronic signal at considerable depths (up to several hundred kilometres), devoid of pejorative features of electromagnetic wave propagation.

State-of-the-art, usable ELINT systems should offer three important functionalities. The first one is the precise determination of technical parameters, which include, among others, carrier frequency of the signal pulse and duration of the pulse, and in ad hoc recognition, signal amplitude [8]. The mentioned process should be performed during signal acquisition in quasi-real time. The second important functionality is the development of information concerning the location of signal emission sources by correlation and analysis of recorded data regarding the technical parameters of signals, characteristics of antennas used, and telemetry data of the UAV platform, defining each of its six degrees of freedom. Within the described functionality, the result of data processing should be visualised on a digital map in the form of agreed symbols along with information about a degree of risk [9]. The graphical user interface should guarantee a positive user experience (UX), determining the high intuitiveness of the system and its ease of use, which will result in minimising the time of decision-making and then operational activities, such as the destruction of detected signal emission sources located on the battlefield. The third important functionality of modern ELINT systems is the recognition of emission source type and, in special cases, the identification of a specific emission source based on the processing of reference data using AI (artificial intelligence) algorithms that analyse multidimensional feature spaces [10]. An important fact is that in the area of operational activities in the electromagnetic environment, current ELINT devices and systems do not have advanced mechanisms that would enable the pattern recognition process and classification of particular copies of emission sources belonging to a set of devices of the same type. Typically, the above-mentioned recognition and classification processes involve a decision regarding only the type of analysed devices emitting a radar signal. The methods used to distinguish particular copies of emission sources are usually implemented in a post-mission mode and constitute a very sophisticated field in the ELINT area, defined as specific emitter identification (SEI) [11,12,13].

Processing of acquired data and information obtained on their basis constitutes intelligence support from ELINT systems and allows for building information advantage on the modern battlefield. Information advantage should be perceived as domination over an opponent in the information space, which results from the ability to collect, process, and, finally, effectively use data or information [14]. Intelligence support from ELINT systems may involve the use of electronic countermeasures to defend information and exclude all kinds of adversary activities seeking to organise information potential [15]. On the other hand, the described support may concern the provision of situational awareness regarding the state of the battlefield through access to current information. Through such actions, it is possible to adapt available resources to the dynamic conditions of the battlefield during the performance of operational tasks.

ELINT systems typically consist of three main components. The first one is a specialised set of antennas, covering the signal acquisition band in the basic range <0.5 ÷ 18 GHz>. The second element is a specialised microwave receiver with high sensitivity. The last module is software, which offers: data selection and reduction, distinctive feature extraction, quasi-optimal feature vector construction, and their processing. As a result of the above-mentioned operations, the ELINT system, in the decision-making process, returns information about the classification outcome or identification of the emission source [1,2].

In this article, the authors present the pioneering research results, which concern the acquisition and analysis of radar signals from six battlefield radars of the same type. With the utmost care, the authors ensured comparable measurement conditions for each of the radar emission sources used. It should be emphasised that making the above-mentioned measurements is not a trivial task, especially if the tests involve comparing several copies of the same type of radar emission source. In the article, the authors attempt to solve the problem, which has not been a subject of research yet. The mentioned research problem concerned identifying each of the six units of the tested battlefield radars. They were represented in the feature space in the form of data particles [16], created through the extraction of distinctive features and multi-criteria evaluation of artificial intelligence algorithms [17].

The data classification process is currently a significant thread carried out as part of the reconnaissance of electromagnetic radiation in the knowledge area of operational activities in the electromagnetic environment on the modern battlefield. Thus, the aim of the article was to determine the effectiveness of developed algorithms in the process of distinguishing particular copies of emission sources, i.e., making specific emitter identifications. Simultaneously, it was undertaken to optimise the aforementioned process under the criterion of maximising the value of the F_macro_ measure. However, apart from the accuracy of the system, special attention should be paid to the time required to carry out the process of reconnaissance and identification of emission sources on the modern battlefield. In this context, the objective function should be to minimise the said time in order to improve the decision-making process regarding, for example, combating detected emission sources. Therefore, the second criterion for optimising the process in question was to minimise the recognition and identification time.

The study, which was divided into two experiments, analysed the data from six battlefield radars. As part of the first stage, the analysis was carried out in a two-dimensional feature space (signal pulse amplitude and pulse duration) using the geometrical divide (GD) algorithm developed by the authors in 2020 [17]. The second experiment was devoted to the analysis of signals described in a three-dimensional feature space (signal pulse amplitude, pulse duration, and pulse carrier frequency). In the second stage of the research, due to the limitations of the GD algorithm, an improved method was used: the hypergeometrical divide (HypGD), which was published in the doctoral dissertation of one of the article authors in 2022 [18].

The authors of this article are currently working on the aforementioned solutions, which in their entirety are groundbreaking works in the field of ELINT recognition on UAV platforms, and the results of these studies will be successively presented in subsequent works.

## 2. Related Technologies and Works

To date, several algorithms have been proposed in the scientific literature that are used to create data particles. 

The most popular algorithm thereof, which, due to its speed, has found application in many practical problems, is the approach that creates a data particle based on the entire class of objects in relation to a cardinality of 1 ÷ 1 (1CT1P). However, the disadvantage of this approach is a significant loss of information about the distribution of indivisible elements—atomic data particles [16].

A similar strategy for creating a data particle occurs in the second approach. A distinction is that a different underlying element is used to create the data particle, which in the second method is the atomic dataset object (1OT1P). However, due to the storage of information about all elements of the set, it is necessary to compare a large number of objects later [16].

Another approach called the maximum distance principium (MDP) creates a data particle based on the criterion of distance between objects described with the same labels [16].

The fourth method in this group is the simulated gravitational collapse (SGC) method, which creates prototypes of classes and solves the problem of classifying datasets in which the distributions of attribute values of objects of individual classes determine the overlapping of elements with different labels in the feature space [19].

The next method belonging to this group, developed by the article authors in 2020 and later updated in 2021, is the algorithm for creating a data particle by its geometrical divide (GD) in a two-dimensional feature space. The approach solves the problem of classifying data sets in which the gravity centres of classes are in close proximity and the objects described with different labels overlap each other at the same time. In addition, the method of data particle geometrical divide is characterised by good efficiency in the problem of presence detection in confined spaces. However, the disadvantage of this approach is that it can only be applied to two-dimensional data sets [17].

The last method occurring in the literature and belonging to the described set is the approach of creating a data particle through its hypergeometrical divide (HypGD). It is an extension of the geometrical divide method, which was published by the authors of this article in 2020 and was the subject of further research presented in 2021. Motivation to develop the hypergeometrical divide algorithm was determined by a significant disadvantage of the GD algorithm, which is the possibility of using it only in the process of classifying data sets whose objects belong to the feature space of dimension ℝ^2^. The Hypergeometrical Divide method eliminates that limitation and makes it possible to create a data particle by its geometrical divide in data sets whose elements belong to the feature space of dimension ℝ^2+^ [18].

## 3. ELINT Data Processing in SEI Module of Proposed System

### 3.1. Overall System Concept

According to the fact mentioned in the introduction of this article, the acquisition of ELINT signals is carried out by an antenna or a specialised antenna set. Looking at the available antennas, it can be noticed that they are characterised by different antenna gain and beam width in elevation and azimuth. The mentioned parameters are further variables as a function of frequency. This is particularly important in the process of defining the emission source azimuth of a particular pulse and then its location. In the proposed system, a horn antenna for outdoor applications was used (A-INFO LB-20180-SFSPO), whose key parameters, significant in the context of tests carried out, are presented in Table 1.

In the proposed SEI module of the ELINT system, received signals are processed by a broadband signal processor built into the broadband analyzer. The selected device is able to record signals in the band from 100 MHz to 18,000 MHz and analyse them in the 500 MHz instant band. The exact technical specification of the equipment used is presented in Table 2.

Based on a combined spectral and time analysis of the received electromagnetic space, the device automatically detects pulses and performs the task of extracting their features. As a result of the implementation of the above-mentioned tasks, a pulse description word (**PDW** feature vector) is built, which describes a single electromagnetic pulse. Taking into consideration the characteristics of the broadband analyzer used, it was assumed that in the proposed SEI module of the ELINT system, each **PDW** vector is described at least by a set of the following features:

- pulse amplitude (AMP),
- pulse duration (PD),
- pulse carrier frequency (RF),
- time of arrival (TOA).

The structure of the applied **PDW** vector is presented in the form of Equation (1).

**PDW** = [AMP, PD, RF, TOA], (1)

Using the principles of designing databases for ELINT purposes described in the literature for the proposed system, a pulse database (PDB) was created for data storage. It uses a flat-file database model; therefore, the PDB consists of only one table, which is called PDW_Vectors. In the proposed SEI module of the ELINT system, the PDB database is stored in the form of a text file in.csv format, in which the first row contains the table column names. Each subsequent line of the file contains a value set describing the single pulse parameters. A semicolon was used as a column separator. It should be noted that using one selected model of data storage, it is not possible to define data types for individual columns. However, the mentioned data comes from a broadband analyzer that guarantees the unification of data types within a single parameter for all pulses. It is worth mentioning that in the event of a need to perform operations on selected data, they may be imported into a selected database management system (DBMS). If there is a necessity to manually edit the data generated by the broadband analyzer, the DBMS in the data processing will perform validation after defining the data types. In order to define the data structure in the DBMS, its graphical interface or the DDL data definition language can be used. The set of attributes in the PDW_Vectors table consists of the **PDW** vector attribute set, identifier (ID), and label (LABEL). The role of the ID attribute is to enable unique identification of each PDB record. However, the idea of introducing the LABEL column is to give the possibility of assigning information about the emission source of a particular pulse. The scheme of the flat-file database used in the proposed solution is shown in Table 3.

In the developed SEI module of the ELINT system, the assumption is that in the PDB database there is a subset grouping **PDW** vectors with a known label, which constitutes information allowing to identify an emission source that emitted a particular pulse. The existence of this subset is a necessary but not sufficient condition to carry out the identification of emission sources. In order to realise the process, it is possible to use classifiers belonging to the group of supervised machine learning techniques in the area of artificial intelligence. Supervised algorithms apply a priori knowledge in the pattern recognition process. This means that the learning stage is based on a certain training set in which each object is described with specific features and has an assigned label, on the basis of which the system is able to recognise new objects whose label is unknown. This is a hallmark of supervised machine learning methods. In the context of the presented theory, it is assumed that **PDW** vectors with unknown labels are recorded during reconnaissance activities. Then, in quasi-real time, each of them is subjected to the process of comparison with PDB records, which have non-empty values for the LABEL attribute. The comparison operation is a key stage of the classification process, which is carried out by the proposed ELINT system. The purpose of carrying out those activities is to determine the labels for registered **PDW** vectors. These labels determine a particular copy of the emission source with a non-zero probability.

### 3.2. Application of Data Particle Geometrical Divide Algorithms in Radar Signal Recognition

The idea of using the data particle geometrical divide is to obtain the structure of a learning set that will enable balancing the performance of the classification algorithm. This optimisation is carried out based on two criteria. It concerns enabling the predictive method to achieve high accuracy in a short time. This is particularly important in electronic reconnaissance systems operating in dynamic battlefield conditions, when a large amount of data is recorded. Rapid data processing is a critical process in the context of a constant need to support command and operational activities.

An advantage of selected algorithms for creating data particles by their geometrical divide is the fact that those approaches cope well with the analysis of the feature space, in which there is no clear separation of atomic data particles. The aforementioned phenomenon is an immanent attribute of the feature space, which contains data describing the pulses coming from different emission sources of the same type. The correlation between these two facts is based on the premise that the use of data particle geometrical divide methods will positively affect the efficiency of classification and identification processes.

The idea behind the approaches to creating data particles through their geometrical divide is to manipulate the affiliation of atomic data particles to particular data particles. It is done by iteratively decomposing each particle into two new data particles. As a result, masses of data particles and the location of their central points change, which ultimately affects the force values with which the existing data particles act on the newly classified samples. That also influences the decision boundaries, which directly alter the efficiency of a classifier.

In order to divide a data particle, it is necessary to determine to which of the two newly created data particles the atomic data particles forming the processing data particle will be assigned. For this purpose, the first step in both algorithms (geometrical divide [17] and hypergeometrical divide [18]) is to define the data particle divide depth level. Then, in an iterative manner, until the assumed level of divide depth is reached, the existing data particles will be subjected to decomposition procedures. The first two steps of the data particle divide operation—determination of the geometric centre and the centre of mass—are common to both methods. Then, for each data particle, it is essential to check whether defined centres do not overlap. If yes, the algorithms will terminate, and the feature space will contain the data particles created in the previous divide iteration. However, if the centres are not equal, the methods shall go to the next stage of determining the object dividing the data particles, which is different for each algorithm and consists in:

- Geometrical Divide: defining a line equation passing through the geometric centre and the centre of mass of the data particle processed;
- Hypergeometrical Divide: defining the equation of a hyperplane by determining the normal vector components of the searched hyperplane by subtracting the coordinates of the centre of mass from the coordinates of the geometric centre.

**Figure 1:**
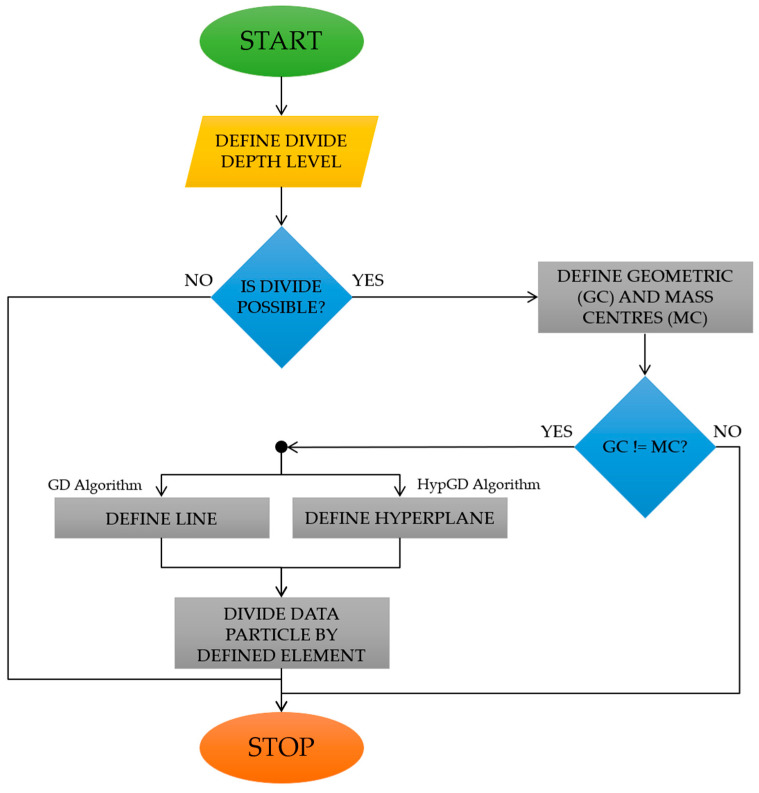
Flowchart representing overall applied algorithm idea (source: own elaboration based on [17,18]).

The last stage of data particle divide, which is common to both methods, is to check the position of each atomic data particle in relation to the object used for divide, and on this basis, assign an atomic data particle to one of the two newly created data particles [17,18]. The described algorithm idea is visualised in Figure 1.

### 3.3. Evaluation Method and Quality Metrics

At the current stage of research and development work on the SEI module of the ELINT system, the key issue is learning its identification capabilities. When performing the architectural decomposition of the project in question in the aforementioned context, the effectiveness of applied artificial intelligence algorithms becomes the central point of interest. Therefore, tools and methods used in publications on artificial intelligence algorithms have been applied in the research.

In order to avoid overfitting of applied algorithms and correct assessment of their generalisation properties, the k-fold cross-validation method was used in the process of evaluating the concept of the SEI module of the ELINT system. The k parameter was assigned a value k = 10. In each iteration, the method, parameterized this way, divided the entire data set into two subsets:

- test subset, which constituted 10% of the set;
- training subset, which includes the remaining 90% of the original set samples.

The results of the identification process were collected in the form of a confusion matrix containing the following elements:

- TP—true positive;
- TN—true negative;
- FP—false positive;
- FN—false negative.

To describe the quality of the identification process, F_macro_ measure was chosen, as described by Equation (2).

(2) $$F_{macro}=2\cdot {PRECISION}_{macro}\cdot {RECALL}_{macro}\cdot {\left({PRECISION}_{macro}+{RECALL}_{macro}\right)}^{-1},$$

Determining the value of the F_macro_ measure should be preceded by defining the values of the PRECISION_macro_ and RECALL_macro_ measures. Equation (3) describes PRECISION_macro_. According to the definition, it is the arithmetic mean of precision values for each class. In the problem of identifying emission sources, a class should be understood as a single copy of an emission source belonging to an n-element set.

(3) $${PRECISION}_{macro}=(\sum_{i=1}^{n}{TP}_{i}\cdot {\left({TP}_{i}+{FP}_{i}\right)}^{-1})\cdot n^{-1},$$

The value of the RECALL_macro_ measure, which is expressed in Equation (4), is defined in an analogous way.

(4) $${RECALL}_{macro}=(\sum_{i=1}^{n}{TP}_{i}\cdot {\left({TP}_{i}+{FN}_{i}\right)}^{-1})\cdot n^{-1}$$

### 3.4. Data Acquisition and Pre-Processing

For the purposes of planned experiments, signals from six battlefield reconnaissance radars of the same type were recorded with the application of an antenna and a broadband analyzer. The broadband analyzer saved each pulse in the form of a PDW vector. A set of three features describing the measured radar signals were taken into account in the study. The set included:

- AMP—pulse amplitude [dB];
- PD—pulse duration [ns];
- RF—pulse centre frequency [MHz].

**Figure 2:**
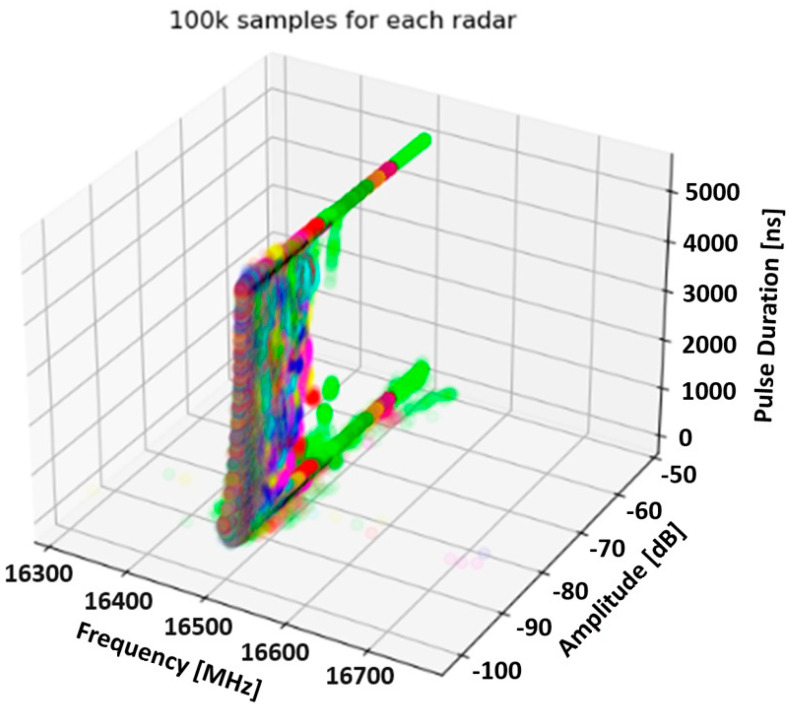
Three-dimensional feature space built on the basis of a data set containing approx. 100,000 PDW vectors for each copy of the emission source. The same colour points refer to the same instance of radar (source: own elaboration).

In total, about 9 million PDW vectors have been collected—less than 1.5 million for each copy. At the beginning of the analysis, an attempt was made to illustrate the created database in the form of a three-dimensional feature space. The above-mentioned activity showed that there is no separation between the PDW vectors coming from different copies of an emission source of the same type; a significant majority of impulses overlap in the feature space. Then, a down-sampling was performed, as a result of which a new dataset was created, which consists of 100k samples for each radar. The feature space built on the basis of this dataset is shown in Figure 2.

### 3.5. Selection of the Most Informative Feature Subspace

**Figure 3:**
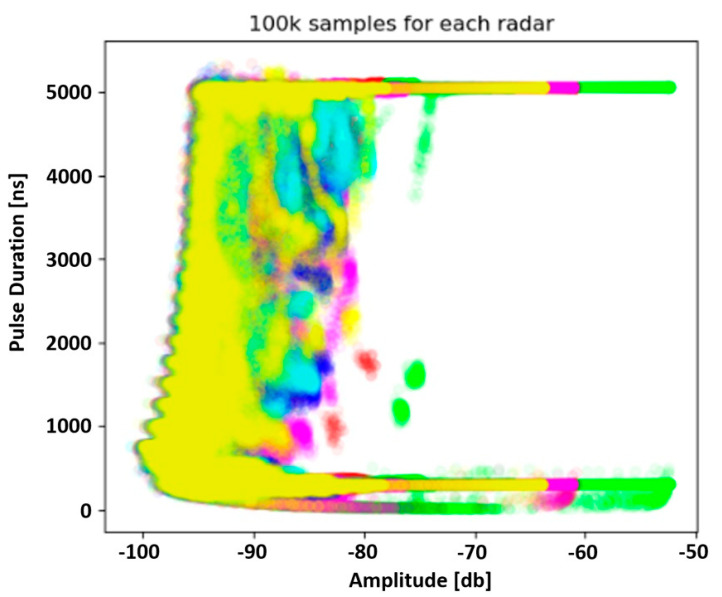
Two-dimensional feature space built on the basis of a data set containing approx. 100,000 PDW vectors for each copy of the emission source. The same colour points refer to the same instance of radar (source: own elaboration).

Analysing the constructed feature space, it was found that the centre frequency of the pulse (FREQ) significantly determines the effect of overlapping objects in the feature space. Therefore, an attempt was made to reduce the dimension of the used data set, which resulted in the construction of a two-dimensional feature space depicted in Figure 3.

**Figure 4:**
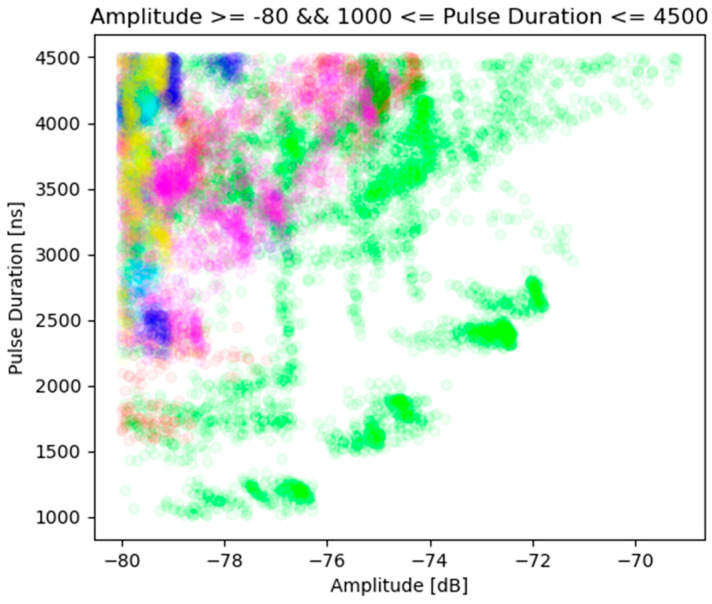
A fragment of two-dimensional feature space containing PDW vectors that meet the following criteria: PD ∈ [1000; 4500] and AMP >= −80. The same colour points refer to the same instance of radar (source: own elaboration).

**Figure 5:**
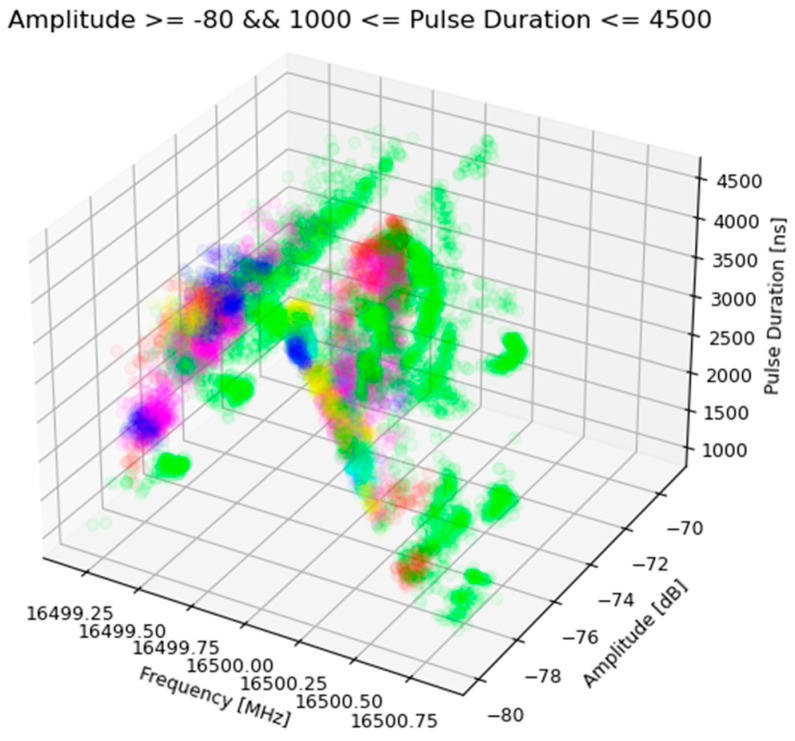
A fragment of three-dimensional feature space containing PDW vectors that meet the following criteria: PD ∈ [1000; 4500] and AMP >= −80. The same colour points refer to the same instance of radar (source: own elaboration).

Looking at Figure 3, it can be observed that there is an area of the feature space in which there is a certain separability of pulses coming from different copies of the tested emission source. It can also be seen that the pointed area consists of PDW vectors describing pulses whose pulse duration is in the range of 1000 ns to 4500 ns and whose amplitude exceeds −80 dB. Figure 4 shows the visualisation of the described fragment of the feature space, while Figure 5 shows the same fragment of the feature space, extended by one dimension—the centre frequency of the pulse.

## 4. Results

### 4.1. Identification Based on Two-Dimensional Feature Space

**Figure 6:**
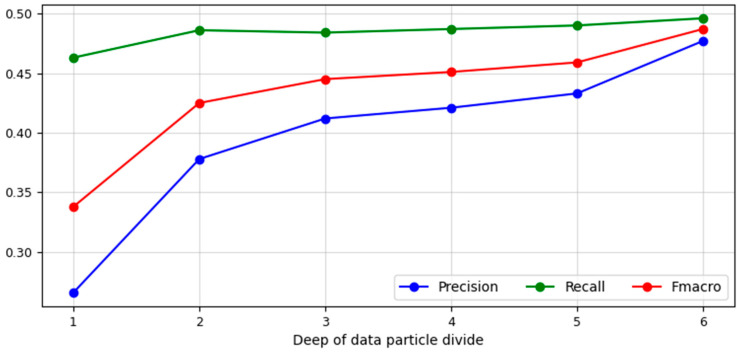
Values of precision, recall, and F_macro_ measures obtained using the Geometrical Divide algorithm at particular levels of the depth of data particle divide (source: own elaboration).

During the first experiment, six copies of the battlefield reconnaissance radar were identified by analysing a set of data describing two operating parameters of the aforesaid devices: amplitude and pulse duration. In the pattern recognition process, one of the new artificial intelligence algorithms, geometrical divide, was used. Figure 6 presents the obtained values of three measures defining the quality of identification.

The values describing the depth level of the data particle divide applied in the Geometrical Divide algorithm were placed on the *X*-axis. Whereas the values of measures: precision, recall, and F_macro_ were located on the *Y*-axis. As can be seen from Figure 6, after dividing the data particles at the depth level d = 1, a precision of 0.266 was obtained, while for the recall measure, a level of 0.463 was reached, which influenced the value of the F_macro_ measure, which amounted to 0.338. After another division of the existing data particles, an increase in the value of all the listed measures was obtained in the recognition process. The precision and recall values increased to 0.378 and 0.486, respectively. In consequence, F_macro_ value was 0.425. After the third iteration of data particle division using the geometrical divide method, an increase in identification precision was obtained, the value of which reached 0.412, while the recall value decreased by 0.002, reaching 0.484. With this state of precision and recall, the value of the F_macro_ measure was 0.445. On the grounds of the implementation of the fourth iteration of data particle division, the applied algorithm obtained a change in precision, which increased to 0.421. In the case of recall, the improvement result changed the measure value to 0.487. As a result of the described iteration of data particles, the value of the F_macro_ measure was obtained at the level of 0.451. The data subjected to the recognition process, obtained as a result of data particles being divided at the depth level d = 5, determined that precision and recall of the geometrical divide algorithm were at the levels of 0.433 and 0.490, respectively, which influenced the value of the F_macro_ measure, which this time was 0.459. Due to the implementation of data particle division at the maximum depth level for the processed data set, a precision of 0.477, a recall of 0.496, and F_macro_ measure of 0.487 were obtained. The distribution of atomic objects in the feature space determined that it was not possible to make a divide at successive levels of depth. The last value of the F_macro_ measure was the maximum value, describing the quality of the geometrical divide algorithm in the process of identifying six copies of the emission source based on the analysis of pulse duration and amplitude. 

**Figure 7:**
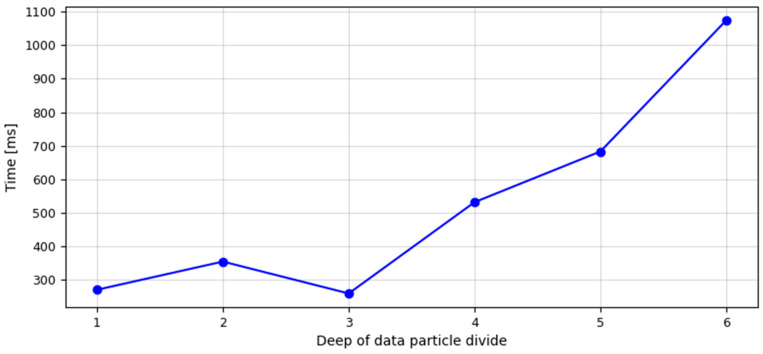
Function of the identification process time (expressed in milliseconds) in the domain of data particle divide depth level for the Geometrical Divide algorithm (source: own elaboration).

On the modern network-centric battlefield, apart from maximising the effectiveness of actions, an important issue is also minimising the time of the recognition and identification process. Therefore, the second part of the experiment is an analysis of the time taken for the Geometrical Divide algorithm to complete the identification process. Figure 7 visualises the time function of the identification process in the domain of the data particles divided by the depth level.

The values describing the depth level of the data particle divide applied in the Geometrical Divide algorithm were placed on the *X*-axis. On the other hand, the *Y*-axis contains the values describing the identification process time, which was expressed in milliseconds. Analysing the figure, it can be observed that based on the data obtained by dividing them at the depth level d = 1, the identification process lasted 271 ms. By making the next divide at the depth level d = 2, and using the data particles created this way, the pattern recognition process time was extended to 355 ms. Using, in the identification process, the data obtained as a result of data particle division at the depth level d = 3, the time of this process was 260 ms. Application of the data set obtained by dividing at the depth level d = 4 extended the identification process time to 532 ms. In addition, using the data particles created as a result of their division at the depth level d = 5, the identification process was realised in 683 ms. At the maximum level of the data particle divide depth for the processed data set, d = 6, the geometrical divide algorithm completed the identification process in 1074 ms. The last value defines the minimum data processing time needed to obtain the maximum effectiveness of the geometrical divide algorithm in the identification process of six radar copies, based on the analysis of pulse duration and amplitude.

As part of this experiment, a performance test of the k-nearest neighbours (k-NN) algorithm and the naive Bayes (NB) method known in the literature was also conducted. By processing the two-dimensional feature space, consisting of the dimensions pulse amplitude and pulse duration, the F_macro_ measure value was obtained at the level of 0.551 in 54485 ms by the k-NN approach. However, for the NB algorithm, the F_macro_ measure at the level of 0.331 and the classification time of 1513 ms were obtained.

### 4.2. Identification Based on Three-Dimensional Feature Space

**Figure 8:**
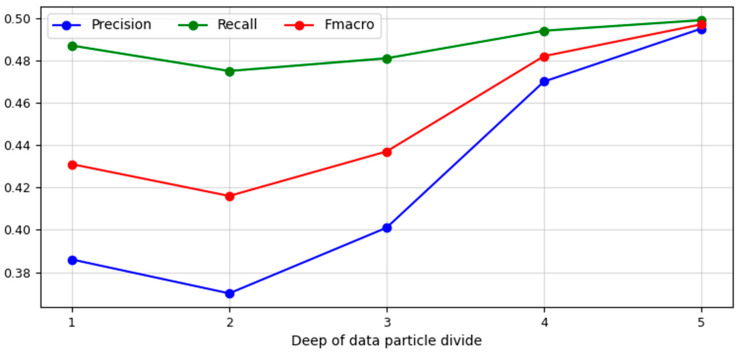
Values of precision, recall, and F_macro_ measures obtained using the Hypergeometrical Divide algorithm at particular levels of the data particle divide depth (source: own elaboration).

In the second experiment, a data set describing six copies of an emission source of the same type was recognised and identified. That data set consisted of three operating parameters: frequency, pulse duration, and amplitude. It determined the necessity of using the hypergeometrical divide algorithm because the geometrical divide method cannot be used for a data set described in a three-dimensional feature space. Figure 8 presents the obtained values of three quality measures describing the recognition/identification process.

The values describing the depth level of the data particle divide applied in the Hypergeometrical Divide algorithm were presented on the *X*-axis. The values of the following measures: precision, recall, and F_macro_ were located on the *Y*-axis. As can be seen, in consequence of the first divide of data particles (depth of data particle divide d = 1), a precision of 0.386 and a recall of 0.487 were obtained, which influenced the value of F_macro_ measure, which was 0.431. After another division of the existing data particles, there was a decrease in all the measures mentioned. Precision and recall values decreased to 0.370 and 0.475, respectively. Resultingly, F_macro_ value was 0.416. In subsequent iterations of the data particle divide, an increase in all applied quality measures was noted. In the third iteration of the hypergeometrical divide method, a significant increase in identification precision was obtained, the value of which was 0.401, while the recall value in the third iteration of the algorithm increased by 0.006, reaching 0.481. The result of the presented state of affairs was the fact that the value of F_macro_ measure amounted to 0.437. Due to the implementation of the fourth iteration of the data particle divide, the algorithm returned the results, in which the precision value again recorded a significant increase and amounted to 0.470. In the case of recall, the improvement in results returned a value of 0.494. For the described iteration of the data particles, the F_macro_ value reached 0.482. In the last iteration of the data particles divided by the hypergeometrical divide method, almost identical values of precision (0.495) and recall (0.499) were obtained, which finally allowed us to obtain an F_macro_ value equal to 0.497, which was the maximum value describing the quality of the hypergeometrical divide algorithm in the process of identifying six radar copies of the same type based on frequency, pulse duration, and amplitude. The distribution of atomic objects in a feature space determined that it was not possible to make a divide at subsequent levels of depth.

**Figure 9:**
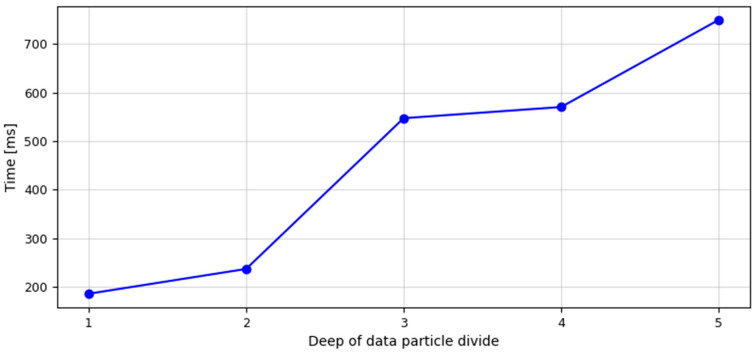
Function of the identification process execution time (expressed in milliseconds) in the domain of the data particle divide depth level for hypergeometric divide algorithm (source: own elaboration).

As mentioned in the section devoted to experiment number one, in the process of recognition or identification, in addition to maximising the effectiveness of the algorithm, the time of data processing is also an important property. Due to this fact, the course of the function of identification process time in the domain of data particle divide depth level was visualised in Figure 9.

The values describing the depth level of the data particle divide applied in the geometrical divide algorithm were placed on the *X*-axis. Whereas, the *Y*-axis contains the values describing the identification process time, which was expressed in milliseconds. Looking at the graph, it can be seen that based on the data obtained by dividing them at the depth level d = 1, the identification process lasted 186 ms. By making another divide at the depth level d = 2 and using the data particles created this way, the pattern recognition process was extended to 237 ms. Using, in the identification process, the data obtained by data particles divided at the depth level d = 3, the time of this process was 547 ms. The data set obtained by subsequent division (d = 4) extended the identification process to 570 ms. On the other hand, the use of data as an effect of the data particle divide at the depth level d = 5 translated into the implementation of the identification process by the applied algorithm in 749 ms.

The feature space used in this experiment was also processed by the k-Nearest Neighbours (k-NN) algorithm and the naive Bayes (NB) method. By analysing the pulse amplitude, pulse duration, and pulse frequency, the k-NN obtained the F_macro_ value of 0.596, and the implementation time of the classification process was 53,871 ms. The processing of the same feature space by the NB algorithm returned F_macro_ measure value at the level of 0.363 and lasted for 1489 ms.

## 5. Conclusions

The analysis of the obtained results showed that in the problem of specific identification of emission sources based on the processing of **PDW** vectors, a distinctive feature subspace exists in the feature space. Its automatic/quasi-automatic search methods have not been proposed in the literature so far. At present, an effective approach is the manual analysis of recorded data visualisations.

In the process of specific emitter identification based on the analysis of carrier frequency and pulse amplitude, the use of the Geometrical Divide algorithm improves the quality of pattern recognition by 2.922 times compared to the base result obtained randomly, which for six copies is F_macro_ = 1.667. The mentioned improvement in results was achieved in 1074 ms.

In the process of specific emitter identification based on a two-dimensional feature space, the k-nearest neighbours (k-NN) algorithm is 13.1% more effective than the geometrical divide (GD) method. However, the GD method is more than 50 times faster than the k-NN approach. In specific emitter identification from among six copies of the same type, the geometrical divide approach is 47.1% more effective and 29% faster than the Naive Bayes classifier. 

The realisation of the process of specific emitter identification based on the analysis of pulse carrier frequency, pulse duration, and pulse amplitude using the Hypergeometric Divide algorithm improves the quality of pattern recognition by 2.976 times. This quality increase was achieved in 749 ms.

In the case of specific emitter identification based on the three-dimensional feature space, the k-Nearest Neighbours (k-NN) algorithm achieves 19.9% higher efficiency than the Hypergeometric Divide (HypGD) method. Moreover, the HypGD approach performs recognition in over 71 times less time than the k-NN algorithm. In the process of specific emitter identification from among six copies of the same type, the hypergeometrical divide approach is 36.9% more effective and 49.7% faster than the naive Bayes algorithm. 

The data particle creation algorithms, through their geometrical divide geometrical divide and hypergeometrical divide demonstrate good efficiency in the implementation of specific emitter identification processes. As the level of data particles divided by depth increases, the difference between precision and recall decreases. 

An important direction for further research, and thus research problems, may be the issue of determining whether it is possible to automate the process of selecting the most informative feature subspace for the purposes of specific identification of emission sources. An affirmative answer to this question determines further unresolved issues: the definition of optimisation criteria for such algorithms and the design of the mentioned methods.

Another direction in the development of the concept may be the use of an unmanned aerial vehicle as an antenna carrier and a broadband analyzer. It is hypothesised that the indicated strategy would contribute to a significant increase in the distance at which it would be possible to carry out specific identification of radio-electronic emission sources, and thus the entry of ELINT systems to a significantly higher level of functional characteristics.

The next direction of research may be to compare the tested methods with other supervised machine learning algorithms found in the literature. It is recommended that the evaluation be guided by two functions: maximising the accuracy of the identification process and minimising its execution time. These are important features of ELINT systems dedicated to use on UAV platforms.

The last of the proposed research directions may be the development of a proposed system concept with a ground station module for the analysis of collected data. Its purpose would be to perform offline post-mission data processing, including spectral analysis. The idea of this component would be to evaluate the online identification process and develop new patterns for implementation in subsequent reconnaissance activities.

## Figures and Tables

**Table 1:** Significant technical parameters of the antenna used in the tests (source: A-INFO Technical Specification of LB-20180-SFSPO).

Parameter	Value
Frequency Range (MHz)	2000.0–18,000.0
Gain (dB)	12 Typ.
Gain for Frequency 16,500.0 MHz (dBi)	16.6
3 dB Beamwidth Elevation Plane (deg)	85–19
3 dB Beamwidth Horizontal Plane (deg)	77–18
3 dB Beamwidth Elevation Plane for Frequency 16,500.0 MHz (deg)	25.48
3 dB Beamwidth Horizontal Plane for Frequency 16,500.0 MHz (deg)	18.88
Size (mm)	103.8 × 77.9 × 127

**Table 2:** Significant technical parameters of the selected broadband analyzer (source: Website of SeCom Sp z o.o.).

Parameter	Value
Frequency Range (MHz)	100.0–18,000.0
Instant Band (MHz)	>= 500
Noise Factor (dB)	<= 18
Tuning Step (MHz)	312.5
Tuning Time (µs)	50
Pulse Duration Range (ns)	50–1,000,000
PDW Generator Efficiency (PDW/s)	1,000,000
Size (mm)	205 × 190 × 67
Weight (kg)	<= 2.6
Operating Temperature (°C)	from −30 to +50
Temperature Limit (°C)	from −40 to +60
Humidity (%)	up to 95

**Table 3:** Scheme of the PDW_Vectors flat-file database (source: own elaboration).

PDW_Vectors		
ID	INTEGER	NOT NULL
AMP	DOUBLE	NOT NULL
PD	DOUBLE	NOT NULL
RF	DOUBLE	NOT NULL
TOA	INTEGER	NOT NULL
LABEL	TEXT	NULL

## Data Availability

Data are available on request due to military nature restrictions.
